# Supplementary material for: The combination of active partitioning and toxin-antitoxin systems is most advantageous for low-copy plasmid fitness
Source: Nat Commun. 2025 Aug 1;16:7078. doi: 10.1038/s41467-025-62473-8 (PMC12317039; doi:10.1038/s41467-025-62473-8)
Supplement: Supplementary file 2 — Description of Additional Supplementary Files [file 41467_2025_62473_MOESM2_ESM.pdf]

Title: Data S1. Full list of identified partitioning systems in the plasmid dataset.

Description: This dataset contains a list of partitioning proteins along with associated metadata. Partitioning systems were identified in a plasmid dataset from *Escherichia, Salmonella* and *Klebsiella* strains.

Title: Data S2. Full list of toxin-antitoxin systems for screening.

Description: This dataset contains the list of toxin-antitoxin proteins/RNAs from TADB, which were used to screen a plasmid dataset from *Escherichia, Salmonella* and *Klebsiella* strains to identify potential TA loci.

Title: Data S3. Conserved syntenic blocks (CSBs) in X3 plasmids.

Description: The dataset contains information on the CSB containing the *agrB-dqlB* locus in PTU-X3 plasmids.

Title: S4. Oligonucleotides used in this study.

Description: Names and sequences of the oligonucleotides used as PCR primers in this study.
